# Zika Virus MB16-23 in Mosquitoes, Miami-Dade County, Florida, USA, 2016

**DOI:** 10.3201/eid2404.171919

**Published:** 2018-04

**Authors:** John-Paul Mutebi, Holly R. Hughes, Kristen L. Burkhalter, Linda Kothera, Chalmers Vasquez, Joan L. Kenney

**Affiliations:** Centers for Disease Control and Prevention, Fort Collins, Colorado, USA (J.-P. Mutebi, H.R. Hughes, K.L. Burkhalter, L. Kothera, J.L. Kenney);; Miami-Dade County Mosquito Control, Miami, Florida, USA (C. Vasquez)

**Keywords:** Aedes aegypti, Miami, Zika virus, isolate, Caribbean, Florida, viruses, mosquitoes, MB16-23

## Abstract

We isolated a strain of Zika virus, MB16-23, from *Aedes aegypti* mosquitoes collected in Miami Beach, Florida, USA, on September 2, 2016. Phylogenetic analysis suggests that MB16-23 most likely originated from the Caribbean region.

In 2016, outbreaks of locally transmitted Zika virus occurred in Miami (Wynwood neighborhood) and Miami Beach, in Miami-Dade County, Florida, USA (*1*). During these outbreaks, a Centers for Disease Control and Prevention (CDC) emergency response team was deployed to assist Miami-Dade County disease surveillance and control efforts. CDC entomologists within the CDC emergency response team worked with Miami-Dade County Mosquito Control and sampled mosquito populations using BG-Sentinel type-2 traps (Biogents AG, Regensburg, Germany) to determine basic entomological parameters. Routinely, mosquitoes were collected, identified to species on the basis of the morphologic characteristics described by Darsie and Ward (*2*), and shipped inactivated and preserved in RNAlater (Ambion Inc., Austin, TX, USA) to the Bronson Animal Disease Diagnostic Laboratory (Kissimmee, FL, USA) for Zika virus testing.

In addition to the routine outbreak protocol, 2 BG-Sentinel type-2 traps were placed at a construction site near the intersection of James Avenue and Lincoln Road (25°47′25.68″N, 80°07′50.24″W) in Miami Beach on September 1, 2016. This site was selected because it was adjacent to a site where Zika cases had been detected. On September 2, 2016, the mosquitoes captured were frozen and shipped on dry ice to the CDC laboratory in Fort Collins, Colorado, USA. In the laboratory, the mosquitoes were identified to species on chill tables; female *Aedes aegypti* mosquitoes were separated into pools of 50 mosquitoes or less. A total of 293 female *Ae. aegypti* mosquitoes were collected (146.5/trap/day), grouped into 7 pools, and processed for presence of arboviral agents by cytopathic effect (CPE) assay.

We triturated pools of mosquitoes in 500 μL of Dulbecco’s modified Eagle medium complete with penicillin (100 U/mL), streptomycin (100 mg/mL), 20% fetal bovine serum, and 50 μg/mL amphotericin B. We used the clarified supernatants from triturated mosquito pools to inoculate Vero (mammalian) cells in 24-well plates. We observed the inoculated cells daily and harvested them upon the appearance of CPE. Of the mosquito pools processed, only 1 pool of 50 female *Ae. aegypti* mosquitoes caused CPE. Final titration of the Vero passage was 7.02 log_10_ PFU/mL. We reinoculated the harvested supernatant onto *Ae. albopictus* C6/36 cells; these cell cultures were maintained at 28°C with complete Dulbecco’s modified Eagle medium supplemented with 2% fetal bovine serum and penicillin/streptomycin. We extracted viral RNA from 140 μL of the supernatant harvested from the C6/36 cell cultures using a QIAamp RNA Mini Kit (QIAGEN, Valencia, CA, USA). We performed reverse transcription PCR confirmation on the extracted RNA using flavivirus-specific primers, as described previously (*3*).

We performed all procedures using commercial products according to the manufacturer’s protocols unless otherwise noted. We generated cDNA from extracted RNA using the NuGEN Ovation RNA-seq system V2 (NuGEN Technologies, San Carlos, CA, USA). Libraries were constructed using the Ion Xpress Plus gDNA Fragment Library preparation kit (Life Technologies, Carlsbad, CA, USA) by fragmenting cDNA for 1.5 min, generating fragments of 250 bp on average; we quantified the libraries using the Ion Library TaqMan Quantitation kit (Life Technologies).

We performed whole-genome sequencing on the Ion Torrent Personal Genome Machine system (Life Technologies) and completed preparation of template-positive ion sphere particles using the Ion OneTouch 2 system and Ion PGM Hi-Q OT2 Kit (Life Technologies). We loaded Ion spheres into an Ion 316 Chip v2 (Life Technologies) and sequenced them on the Ion Torrent Personal Genomics Machine instrument using the Ion PGM Hi-Q sequencing kit (Life Technologies). We generated full genome sequences using a templated assembly in SeqMan NGen (DNASTAR, Madison, WI, USA) and using Zika virus strain PRVABC-59 (GenBank accession no. KX377337) as a reference. We subjected consensus genomes generated by templated assemblies to BLAST analysis (http://www.ncbi.nlm.nih.gov/BLAST) and determined that they were >98% similar to the respective reference sequences.

We aligned the full genome sequence of MB16-23 (GenBank accession no. MF988743) with available sequences in the National Center for Biotechnology Information database (NCBI Bioprojects PRJNA342539 [*4*] and PRJNA344504 [*5*]) using MUSCLE (*6*) on the Cipres Science Gateway (*7*). We performed maximum-likelihood inference with GTRCAT majority rule criterion bootstrapping using RAxML-HPC2 on XSEDE of the Cipres Science Gateway (*8*). We edited output trees with FigTree version 1.4 (http://tree.bio.ed.ac.uk/software).

Phylogenetic analysis showed that MB16-23 was closely related to 9 other sequences from Miami, suggesting a common origin to all these sequences (Figure), and was also closely related to the sequence DominicanRepublic_KY014300, from Santo Domingo, Dominican Republic. This finding suggests that MB16-23 and the 9 related sequences originated from a strain or strains introduced from the Caribbean region. Our results support previous observations that genomes collected in Miami-Dade County during July 2016–November 2016 share a common ancestor with genomes localized to the Caribbean area, particularly the Dominican Republic (*4**,**5*). 

**Figure:**
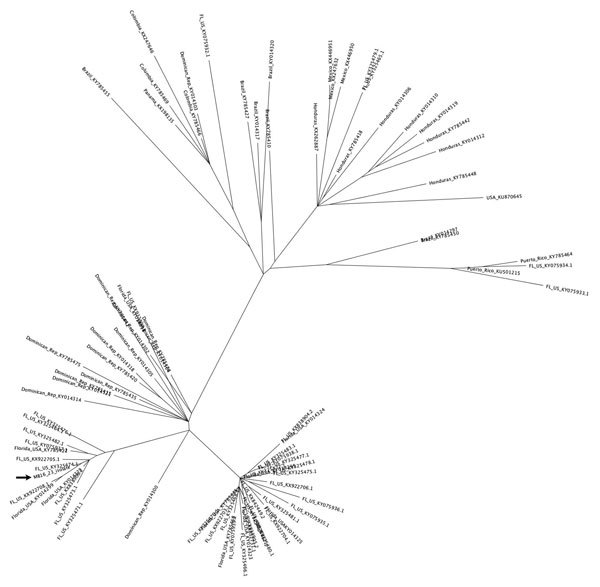
Maximum-likelihood tree generated from the whole-genome nucleotide sequences of 89 strains of Zika virus from Florida, Central America, and the Caribbean. Arrow indicates strain MB16-23, identified in Miami Beach, Florida, USA.

In summary, we report an isolate of Zika virus, strain MB16-23, from a pool of 50 *Ae. aegypti* mosquitoes collected in Miami Beach, Florida. Phylogenetic analysis suggests that MB16-23 shares a common ancestor with other Florida Zika virus genomes as well as genomes localized to the Caribbean region.
